# Protection against cystic echinococcosis in sheep using an *Escherichia coli*-expressed recombinant antigen (EG95) as a bacterin

**DOI:** 10.1017/S0031182022001457

**Published:** 2023-01

**Authors:** Charles G. Gauci, David J. Jenkins, Marshall W. Lightowlers

**Affiliations:** 1 Faculty of Veterinary and Agricultural Sciences, Melbourne Veterinary School, University of Melbourne, Werribee, Victoria 3030, Australia; 2 Charles Sturt University, School of Animal and Veterinary Sciences, Wagga Wagga, New South Wales 2678, Australia

**Keywords:** Bacterin, *Echinococcus granulosus*, EG95, vaccine, whole cell lysate

## Abstract

The EG95 recombinant vaccine is protective against cystic echinococcus in animal intermediate hosts. Preparation of the existing, registered EG95 vaccines involves semi-purification of the vaccine protein, adding to the cost of production. Truncation of the EG95 cDNA, shortening both the amino and carboxy-termini of the protein, leads to high levels of recombinant protein expression. The recombinant EG95 protein was prepared as a bacterin from clarified, whole bacterial lysate, and used in a vaccine trial in sheep against an experimental challenge infection with *Echinococcus granulosus* eggs. The EG95 bacterin was found to induce 98% protection. Use of this in a new generation EG95 vaccine would simplify production, facilitate new sources of the vaccine and potentially enhance uptake of vaccination in control of *E. granulosus* transmission.

## Graphical abstract

**Figure:**
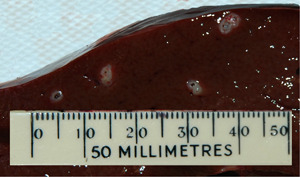

## Introduction

*Echinococcus granulosus sensu stricto* is the aetiological agent of most cases of cystic echinococcosis in humans (Alvarez Rojas *et al*., 2014). The parasite is commonly transmitted by sheep and canines (Cardona and Carmena, 2013). The EG95 vaccine against *E. granulosus* has been found to be highly effective in reducing susceptibility of sheep to an experimental challenge infection with *E. granulosus* eggs (Lightowlers *et al*., 1996, 1999; Lightowlers, 2006), and in reducing the prevalence of cystic echinococcosis in naturally exposed animals (Heath *et al*., 2003; Larrieu *et al*., 2015). The vaccine is now available as a commercial product manufactured in Argentina, as well as China, where it has been implemented as part of a national disease control programme so as to reduce the parasite's transmission and, indirectly, the incidence of human infection (Lightowlers, 2021).

The EG95 recombinant antigen comprises an approximately 16.6 kDa parasite-encoded protein expressed as an amino-terminal fusion with glutathione *S*-transferase (GST) using a pGEX vector in *Escherichia coli*, with the protective responses clearly associated with the EG95 component rather than the GST fusion partner (Lightowlers *et al*., 1996). The EG95 vaccine was initially produced in microbial cultures from bacterial components expressed in soluble form and affinity purified (Lightowlers *et al*., 1996, 1999). Much of the antigen is present in *E. coli*, after induction of recombinant protein, in an insoluble form as bacterial inclusion bodies. A commercial process was developed for use of the insoluble antigen as vaccine (Heath *et al*., 2003). Subsequently, Gauci *et al*. (2011) found that truncation of the original EG95 cDNA, so as to remove short hydrophobic stretches from both the amino- and carboxy-terminal regions of the protein (referred to as EG95NC^−^), greatly enhanced the productivity of the soluble component of the *E. coli*-expressed antigen without affecting its protective efficacy. The truncated EG95 antigen was expressed at a level representing approximately 50% of the total soluble bacterial protein. The potential use of EG95NC^−^ as a bacterin vaccine comprising total soluble protein from lysates of *E. coli* expressing the protein was investigated.

## Materials and methods

The GST fusion of EG95NC^−^ (hereafter referred to as EG95NC^−^) and GST were expressed in *E. coli* BB4 strain, affinity purified using glutathione sepharose and quantified for protein content according to the methods described by Gauci *et al*. (2011). EG95NC^−^ total soluble protein was prepared from induced bacterial cultures by removal of culture medium using centrifugation at 500 ***g***, followed by sonication in phosphate-buffered saline. The bacterial lysate was clarified by centrifugation at 40 000 ***g***, 4°C for 15 min and the soluble protein preparation was filter sterilized through a 0.2 *μ*m syringe filter. The protein concentration was determined (Gauci *et al*., 2011) and the composition of the preparation was estimated as the percentage of EG95NC^−^ in the total soluble protein sample using sodium dodecyl sulphate-polyacrylamide gel electrophoresis (SDS-PAGE) and scanning densitometry (Gauci *et al*., 2011).

A vaccination and challenge trial was undertaken in 30 merino-cross lambs, 4–5 months of age of mixed sex, which were randomly allocated to 3 groups of 10 animals. The trial received Animal Ethics approval from the Australian National University Animal Ethics Committee (F.BTZ.23.08). Each group of vaccinated animals received 2 subcutaneous immunizations 4 weeks apart in the neck with vaccines comprising 1 mg Quil A adjuvant (Brenntag Biosector A/D, Denmark) together with either 50 *μ*g affinity purified EG95NC^−^ protein or the same quantity of EG95NC^−^ as part of the total soluble *E. coli* protein (bacterin). The control group received no vaccination. Two weeks after the second vaccinations, each lamb was given an intraruminal infection with 1000 eggs of *E. granulosus* all derived from the same batch of viable eggs. These eggs were sourced from a naturally infected wild dog obtained from the Local Lands Services of New South Wales, Feral Animal Control Officer through the normal course of the Officer's work. Approximately 1 year after the challenge infection, the animals were humanely euthanized by captive-bolt pistol and the liver and lungs sliced by hand at approximately 3–4 mm intervals to identify *E. granulosus* cysts. Any lesions of unclear origin were examined by histology to confirm the presence of *E. granulosus*. Hydatid cyst viability was determined by excision of the cyst and observation of fluid, protoscoleces or germinal membrane within.

## Results

**Fig. 1.**
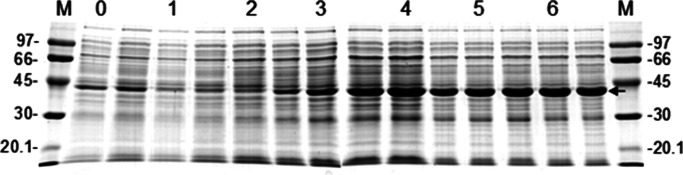
Expression of the GST fusion of EG95NC^−^ in *E. coli* using a laboratory bioreactor (10 litre Eyela fermentor). Each SDS-PAGE lane represents the total soluble protein extracted from samples that were removed from the culture at regular, half hourly intervals. Numbers above gel lanes show the time, in hours, after commencement of the fermentation. Recombinant EG95NC^−^ protein expression was induced by addition of isopropyl-*β*-D-thiogalactoside 3 hours after commencement of the culture. M, Protein markers (kDa). Arrow denotes position of the EG95NC^−^ recombinant protein.

The relative proportion of EG95NC^−^ in total soluble bacterial protein extract used for vaccination was estimated to be 58% (Fig. 1). Scanning densitometry SDS-PAGE analysis of samples removed at regular intervals from an *E. coli* culture expressing EG95NC^−^ showed an increase in the proportion of soluble recombinant protein (relative to *E. coli* proteins) over time, with EG95NC^−^ being the predominant protein present (Fig. 1).

The number of viable *E. granulosus* cysts in individual sheep vaccinated with the antigen obtained from *E. coli* expressing EG95NC^−^, and control sheep are shown in Table 1. One animal in the control group died due to causes unrelated to the experimental procedures. Both affinity purified EG95NC^−^ and non-purified EG95NC^−^ (consisting of total soluble bacterial proteins) induced high levels of protection (94.6 and 97.9%, respectively) in comparison to controls, with no significant difference between the 2 EG95NC^−^groups (Mann–Whitney U test). All control animals developed large numbers of viable cysts following the challenge infection, ranging from 195 to 503 cysts per animal.

**Table 1.** Numbers of viable hydatid cysts in individual sheep following immunization with EG95NC^−^ (plus Quil A adjuvant) and a challenge infection with *E. granulosus* eggs

Group	Number of sheep	Number of viable cysts in individual sheep	Mean	Mean reduction (%)
Non-vaccinated controls	9	195, 300, 307, 341, 386, 418, 447, 454, 503	372	–
EG95NC^−^ affinity pure	10	3, 4, 7, 7, 7, 14, 18, 18, 46, 78	20.2	94.6
EG95NC^−^ total soluble protein	10	0, 0, 1, 3, 5, 8, 9, 13, 15, 26	8.0	97.9

Sheep in test groups were immunized with purified EG95NC^−^ (EG95NC^−^ affinity pure) or crude *E. coli* extract containing EG95NC^−^ (EG95NC^−^ total soluble protein). Cysts present in all animals were macroscopically similar.

## Discussion

The EG95 vaccine produced as either affinity purified antigen, or as total soluble protein from *E. coli*, induced very high levels of protection against a challenge infection with *E. granulosus*. The presence of contaminating bacterial proteins (up to ~50%) did not affect the ability of the EG95NC^−^ antigen to protect vaccinated sheep against *E. granulosus* infection. Heath *et al*. (2003) found that the antigen was also protective as solubilized bacterial inclusion bodies, although the relative purity of EG95 in relation to other bacterial proteins was not indicated. The improved productivity and simplicity of preparation of the EG95 vaccine as a bacterin, comprising total soluble proteins, provides a simple and effective method for producing the vaccine. Based on the bacterial fermentation conditions used here, 1 litre of bacterial culture expressing EG95NC^−^ would produce more than 7000 doses of vaccine. We note that the fermentation conditions had not been optimized and it is likely that an even greater level of antigen productivity would be likely with optimized conditions.

Vaccines comprising whole inactivated *E. coli* bacteria or bacterins are not unusual in the animal health industries (Cox *et al*., 2014); examples include Onderstepoort Biological Products *E. coli* Vaccine for Sheep and Cattle, Elanco's Scour Bos and Zoetis LitterGuard. Hence, use of a bacterin derived from an *E. coli* strain expressing a defined recombinant antigen does not represent an entirely novel vaccine modality. In the vaccine experiment described here, detailed clinical and injection site studies were not undertaken, however, the animals vaccinated with the bacterin had no overt clinical symptoms post vaccination and were not observed to have injection site reactions different to those given the affinity purified EG95 antigen.

Many of the regions where cystic echinococcosis is highly endemic are in relatively poor, developing countries (Deplazes *et al*., 2017), emphasizing the need to minimize vaccination costs. Widespread implementation of vaccination against *E. granulosus* in livestock has the potential to reduce the incidence of human cystic echinococcosis in endemic areas. Our demonstration here of a simple, comparatively inexpensive method for producing the EG95 vaccine may facilitate the vaccine's production in more countries where cystic echinococcosis is highly endemic, contributing to the parasite's control and a reduction in human infections.
